# Linking peripheral low-grade inflammation and blood-cerebrospinal fluid barrier leakage in schizophrenia spectrum disorders – A retrospective analysis

**DOI:** 10.1016/j.bbih.2026.101198

**Published:** 2026-02-18

**Authors:** Timo Jendrik Faustmann, Aykut Aytulun, Armin Bahic, Michaela Jänner, Leonhard Schilbach, Daniel Kamp

**Affiliations:** a Department of Psychiatry and Psychotherapy, Medical Faculty, Heinrich-Heine-University Düsseldorf, Universitätsstraße 1, 40225, Düsseldorf, Germany; b Department of General Psychiatry 2, LVR-Klinikum Düsseldorf, Bergische Landstraße 2, 40629, Düsseldorf, Germany; c Department of Psychiatry and Psychotherapy, University Hospital, Ludwig Maximilians University Munich, Nußbaumstraße 7, 80336, Munich, Germany; d Department of Biometry, LVR-Klinikum Düsseldorf, Bergische Landstraße 2, 40629, Düsseldorf, Germany

**Keywords:** Psychotic disorders, Social isolation, Low-grade inflammation score, Blood-cerebrospinal fluid barrier, Blood-brain barrier, Cerebrospinal fluid, Schizophrenia spectrum disorders, Inflammation

## Abstract

**Background:**

Social impairments and low-grade inflammation (LGI) are associated with psychotic disorders (e.g. schizophrenia spectrum disorders). Social impairments are important symptoms of the disease nonetheless a disturbed social interaction and inflammatory processes are further discussed as being part of the underlying pathophysiology, which is also characterized by blood-cerebrospinal fluid barrier (BCSFB) dysfunction. The relationship between social impairments, peripheral LGI (pLGI) and BCSFB permeability in psychotic disorders, however, is poorly understood. Therefore, we hypothesized that social impairment might be linked to pLGI, which, in turn, might affect BCSFB function in schizophrenia.

**Method:**

We conducted a retrospective chart review of all psychiatric inpatients who underwent lumbar puncture as part of their diagnostic work-up between January 1, 2021, and June 30, 2023 (n = 53). Thirty-one patients diagnosed with SSD (n = 27) or affective psychosis (n = 4) with a C-reactive protein (CRP) serum level <10 mg/L upon admission, indicating the absence of acute inflammation, were included in the analysis.

**Results:**

The cerebrospinal fluid (CSF)/serum albumin ratio – as a measure of BCSFB permeability – was shown to be positively correlated with our measure of pLGI (r = 0.418, p = 0.019) using the pLGI score (previously also named “INFLA-score”, “LGI score”), as well as with age (r = 0.415, p = 0.020) . Additionally, a trend toward a negative correlation with global functioning (GAF) was observed (r = −0.349, p = 0.054).

A multiple linear regression including pLGI, age, and sex yielded the best-fitting model (p = 0.003, corrected R^2^ = 0.337), with all predictors showing independent significant effects.

Interestingly, regarding single parameters of the pLGI score a significant correlation between platelets and the CSF/serum albumin ratio (r = 0.490, p = 0.005) was found. Positive and Negative Syndrome Scale (PANSS6) and social isolation score did not correlate with the model.

**Conclusion:**

These data demonstrate – for the first time – a link between an established peripheral marker of LGI and BCSFB permeability in schizophrenia. Platelets were found to be the main driver of the pLGI score regarding BCSFB permeability. Future research will need to replicate these findings and could explore whether measures of peripheral inflammation could be useful in the diagnostic work-up of patients with psychotic disorders.

## Highlights

• An established score demonstrates peripheral inflammation in schizophrenia.
• Inflammation score correlates with BCSFB leakage in schizophrenia.
• Platelets are driving the peripheral low-grade inflammation score in schizophrenia.

## Abbreviations:

Aβ: Amyloid-β
AI: pathogen-specific antibody indices
BBB: blood-brain barrier
BCSFB: blood-cerebrospinal fluid barrier
CRP: C-reactive Protein
CSF: cerebrospinal fluid
CNS: central nervous system
CT: cranial computer tomography
EEG: electroencephalography
FLAIR: fluid-attenuated inversion recovery
GAF: global assessment of functioning
G/L ratio: granulocyte to lymphocyte ratio
HIV: Human Immunodeficiency Virus
ICD-10-GM Version 2020: International Statistical Classification of Diseases, 10th revision, German Modification, Version 2020
IgA: immunoglobulin A
IgG: immunoglobulin G
IgM: immunoglobulin M
IL-2: interleukin 2
IL-4: interleukin 4
IL-6: interleukin 6
IL-10: interleukin 10
IL-1β: interleukin 1β
LGI: low-grade inflammation
MLR: monocyte to lymphocyte ratio
MRI: cranial magnetic resonance imaging
NICE: National Institute for Health and Care Excellence
NLR: neutrophil to lymphocyte ratio
NMDAR: N-Methyl-D-Aspartate-receptor
OCBs: oligoclonal immunoglobulin bands
PANSS6: short form of Positive and Negative Syndrome Scale
pLGI: peripheral low-grade inflammation
PLR: platelet to lymphocyte ratio
SD: standard deviation
SII: systemic inflammation index
SSD: schizophrenia spectrum disorders
TNF-α: tumor necrosis factor alpha
WBC: white blood cell count

## 1 Introduction

Psychotic disorders are clinically characterized primarily by so-called positive and negative symptoms, as well as (social) cognitive impairments and often take a chronic course (Marder et al., 2019) (Saha et al., 2005). The main therapeutic strategy includes typical and atypical antipsychotics (Meltzer et al., 2008) (Meltzer, 2013) (Faustmann et al., 2025a) (Faustmann et al., 2025b), but further therapeutic strategies and social support strategies are required, because patients often end up in social isolation or are isolated from the beginning of the disease. Further, social deficits could contribute to an activation of immunological pathways and to a status of LGI known to be associated with psychotic disorders (Schilbach, 2016) (Faustmann et al., 2023a) (Faustmann et al., 2023b) (see below).

From an immunological perspective, among patients with psychotic disorders evidence for an innate and adaptive immune response (peripheral and central) has been discussed in the context of an immunological origin, influence or ongoing reaction including prenatal infections and early childhood infections (Brown et al., 2010) (Dalman et al., 2008) reactive microglia, neutrophils, lymphocytes and monocytes, immunoglobulins and cytokines (Mohagheghi et al., 2018) (Steiner et al., 2020) (Miller et al., 2013) (Halstead et al., 2023) (Faustmann et al., 2025c). For some cases, schizophrenia has been discussed as a form of “mild encephalitis” and the term “autoimmune psychosis” has been proposed (Graus et al., 2016) (Bechter, 2013) (Pollak et al., 2020) but antineuronal IgG antibodies (Dalmau et al., 2007) (Endres et al., 2020) are only rarely detected in SSD (Endres et al., 2020) (Endres et al., 2022).

It needs to be questioned what exact role peripheral inflammation (in serum) might be playing in psychiatric diseases. Peripheral immunological reactions have been described as a form of low-grade inflammation (LGI) which is characterized by subclinical inflammatory processes, mainly associated with plasmatic and cellular biomarkers and are also further discussed in cases of cardiovascular diseases and degenerative diseases (Candore et al., 2010) (Danesh et al., 2000; Barbaresko et al., 2013). Interestingly, it has been suggested that a peripheral low-grade immune response could contribute via humoral, neural and cellular transmission to a central nervous immune response that could be potentially relevant for psychotic disorders (Dantzer et al., 2000) (Khandaker et al., 2016) (Halstead et al., 2023).

In psychotic disorders discussions on LGI and its impact via barriers on the central nervous system (CNS) (Bechter et al., 2010) (Rauber et al., 2021) (Moussiopoulou et al., 2025) are interesting approaches. These discussions include two barriers: The BCSFB consists of epithelial cells of the choroid plexus, fenestrated blood vessels and subarachnoid epithelial cells facing the CSF and the blood-brain barrier (BBB) which consists of endothelial cells of vessels, basal lamina, pericytes and astrocytic endfeet and which peripheral inflammatory reactions (humoral, cellular and pathogens) must cross before affecting the CNS. Interestingly, the CSF/serum albumin ratio is a reliable indirect marker of the BCSFB integrity in psychotic disorders and is sometimes incorrect used as marker for BBB integrity even if both barriers show similar functions (Yakimov et al., 2024a) (Moussiopoulou et al., 2025) (Yakimov et al., 2025) (Yakimov et al., 2024b)**.**

From a behavioral perspective, social interaction is an important resilience mechanism and can help to maintain or even improve mental health (Umberson et al., 2010). On the other hand, loneliness can lead to or aggravate symptoms in psychiatric disorders (Stednitz et al., 2006). In psychotic disorders the concept of the vulnerability-stress model was proposed (Zubin et al., 1977). Stress can be seen in a social context and problems in social interaction (Mizrahi, 2016) (MacLeod et al., 2023) or perceived stress due to social capital deficits on an individual, household and area level (Han, 2019). It was discussed that in schizophrenia a social isolation stress can influence neurotransmitter levels and receptor sensitivity (Brandt et al., 2022). In addition to this, negative symptoms increase the risk for social isolation in psychotic disorders as measured by including the areas of Abulia-apathy and Anhedonia-asociality (An der Heiden et al., 2016). Further, in schizophrenia executive functioning and social functioning variance was explained by pro-and anti-inflammatory cytokines like interleukin 6 (IL-6), interleukin 10 (IL-10) and C-reactive protein (CRP). These parameters were discussed to be key factors in predicting social functioning in schizophrenia at a 1-year follow-up compared to type 2 diabetes, depression and bipolar disorder (Gares-Caballer et al., 2022). Beyond this, other behaviors like violence were discussed to be related to inflammation like higher immune cell ratios in patients with schizophrenia (Yu et al., 2024).

CSF diagnostics are recommended as optional in the German national guidelines for schizophrenia and are not mentioned in the National Institute for Health and Care Excellence (NICE) and American Psychiatric Association Practice guidelines (S3-Leitlinie Schizophrenie, 2019) (Psychosis and Schizophrenia in Adults, 2014) (Keepers et al., 2020) and its implementation depends on various factors (e.g. patients’ psychopathology). CSF analysis should be performed to exclude psychiatric manifestations, of a e.g. meningoencephalitis. In light of the obstacles of obtaining CSF-based measures of inflammation, the previously established score of peripheral low-grade inflammation including basic blood results like CRP, white blood cell count (WBC), platelet count and granulocyte to lymphocytes ratio (pLGI score) which was used in e.g. the general population and neurological patients and is an independent risk factor for total mortality (Zhou et al., 2023) (Bonaccio et al., 2016) could be a useful tool in psychotic disorders. In a first approach we wanted to focus on peripheral inflammatory markers (pLGI score) in SSD which present more easily accessible parameters during the diagnostic work-up and its connection towards social deficits and cerebrospinal fluid parameters indicating BCSFB disruption.

## 2 Methods

A retrospective chart analysis was performed for all inpatients of the Department for General Psychiatry 2 at the LVR-Clinic Düsseldorf/Heinrich-Heine-University Düsseldorf, between January 1, 2021, and June 30, 2023. All inpatients, who received a lumbar puncture (n = 53) were screened. Out of this number, n = 31 patients with schizophrenia spectrum disorders (SSD) (n = 27) and affective psychosis (n = 4) were included in the further analysis if CRP serum level upon admission was below 10 mg/l to rule out an acute inflammatory state (according to the pLGI score). The retrospective analysis was approved by the ethics committee of the Heinrich-Heine-University's medical faculty (study number 2023-2513).

**Fig. 1:**
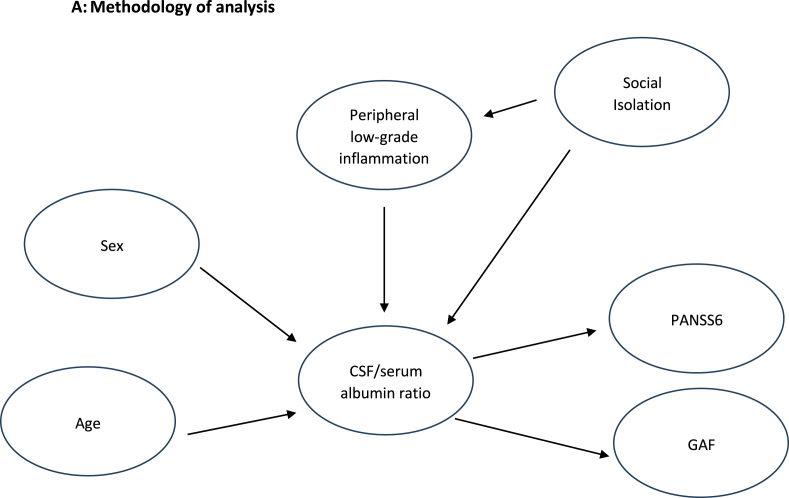

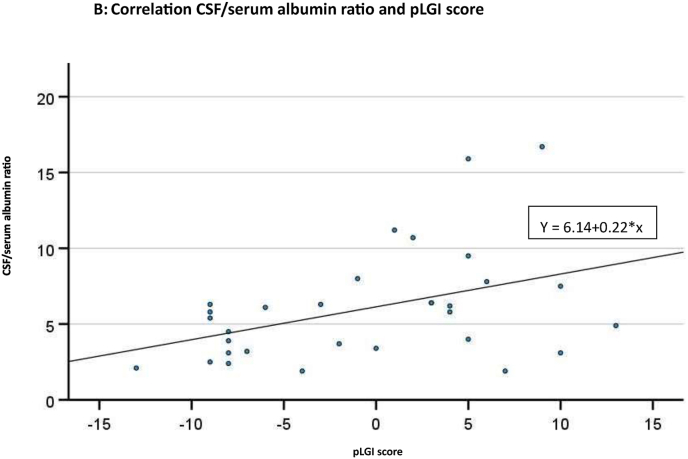
**Fig 1A)** Methodology of analysis. Fig. 1**B)** Correlation of CSF/serum albumin ratio and pLGI score: (r = 0.418, p = 0.019). y = 6.14 + 0.22∗x. N=31 (two patients presented with CSF/serum albumin ratio = 6.4 and pLGI score = 3). CSF: cerebrospinal fluid. GAF: Global Assessment of Functioning. pLGI score: peripheral low-grade inflammation score. PANSS6: short form of the Positive and Negative Syndrome Scale.

We hypothesize that peripheral low-grade inflammation is influencing the CSF/serum albumin ratio and psychotic disorders and that this is further influenced by sex and age. A disturbed social behavior is part of the symptoms of psychotic disorders but additionally to this we focused on missing/disturbed social networks (social isolation score) which influence the disease itself and which we hypothesized are linked to peripheral low-grade inflammation and to alterations of the BCSFB (CSF/serum albumin ratio) Fig. 1A.

### 2.1 Data collection included

#### 2.1.1 Clinical data and blood/urine parameters

International Statistical Classification of Diseases, 10th revision, German Modification, Version 2020 (ICD-10) (ICD-10-GM, 2020), diagnosis, sex, age, migrations status, somatic diseases, Global Assessment of Functioning (GAF) score (Pedersen et al., 2018), previous antipsychotic medication and smoking status were extracted from the charts. Psychopathological findings were evaluated (short version of the Positive and Negative Syndrome Scale (PANSS6)) (Ostergaard et al., 2016). Routine blood parameters like WBC, lymphocyte count, granulocyte count (neutrophils, eosinophils, basophils), granulocyte to lymphocyte ratio (G/L ratio), platelet count, CRP level upon admission, infectious parameter (e.g. Human Immunodeficiency Virus (HIV), Syphilis), urine toxicology screens (cannabinoids, amphetamines, cocaine, opioids, benzodiazepines, 2-Ethylidin-1,5-dimethyl-3,3-diphenylpyrrolidin) were analyzed in the laboratory of our clinic and also extracted. Further, cranial magnetic resonance imaging (MRI) or cranial computer tomography (CT) findings and electroencephalography (EEG) results were extracted from the charts.

#### 2.1.2 Cerebrospinal fluid (CSF) and further blood data

The leucocyte count in CSF was analyzed in our clinic. Protein in CSF, albumin in CSF, albumin in serum, CSF/serum albumin ratio, oligoclonal immunoglobulin bands (OCBs), pathogen-specific antibody indices (AI: including Measles virus, Rubella virus, Herpes simplex virus type 1, Herpes simplex virus type 2, Cytomegalovirus, Varicella-zoster virus, Epstein-Barr virus) were analyzed by an external cooperation partner - Synlab Laboratories Leverkusen, Germany.

Anti-neuronal antibodies against cell surface and intracellular epitopes in CSF and serum (NMDAR, gamma aminobutyric acid A receptor, gamma aminobutyric acid B receptor, IgLON5 receptor, Glycine or Leucine-rich glioma-inactivated 1 protein, contactin-associated protein-like 2, alpha-amino-3-hydroxy-5-methyl-4-isoxazole propionic acid receptor 1/2, dipeptidyl-peptidase-like protein-6, metabotropic glutamate receptor 1 and 5, glutamate decarboxylase, Glycin receptor, Amphiphysin, cronveinten 2/collapsin response mediator protein 5, Ma2/Ta (PNMA2), Ri, Yo, Hu, Recoverin, Sox1, Titin, Zic4, DNER/Tr, Neuropil, glial fibrillary acidic protein, Adenylatkinase-5, anti-neuronal nuclear antibodies 3, Neurexin 3alfa) were analyzed by Laboratory Krone Bad Salzuflen, Germany using diagnostic on mouse brain, cell-based assays and immunoblot.

#### 2.1.3 Scores

An established pLGI score, previously used in studies involving the general population and neurological patients (Bonaccio et al., 2016) (Zhou et al., 2023) (Wang et al., 2023) (Pounis et al., 2016), was applied in this study (some of the studies included the neutrophil to lymphocyte ratio and others the granulocyte to lymphocyte ratio). In order to reduce complexity of the data, the influence of outliers and to avoid strong parametric assumptions we generated deciles of four parameter (CRP, WBC, platelet count, G/L ratio). For the four highest deciles (7 to 10), the score increased from 1 to 4, while the lowest deciles (1 to 4) were negatively scored from −4 to −1. Deciles 5 or 6 were scored 0. Each of the four parameters (CRP, WBC, platelet count, G/L ratio) were included and the pLGI score ranged from between −16 and +16., with higher LGI intensity corresponding to a higher pLGI score. CRP serum level for all patients was below 10 mg/l upon admission (Bonaccio et al., 2016).

A social isolation score was used to assess deficits in social interaction or the social network of patients (Shankar et al., 2011) (Steptoe et al., 2013) (Gao et al., 2024) (Crowe et al., 2021) and was previously used in psychotic patients (Chen et al., 2024). Upon admission in our clinic the social service assessment among others includes questions on living conditions and with who the patient is living, relationship status, if there are relatives, children or pets in need of care by the patient, who is the social reference person of the patient, age of one's own/contact to children and if legal care or assisted living is existing or not. Further, the charts were screened for the admission situation in the clinic (accompanying person) and if visitors were received during the treatment or not. A four-item sum score was generated retrospectively, with one point being assigned for each of the following: (a) not being in a partnership, (b) living alone, (c) having less than monthly contact with family of origin and (d) having less than monthly contact with friends. Higher scores hereby indicate greater social isolation.

Psychopathology was evaluated retrospectively upon admission using the PANSS6 score (Ostergaard et al., 2016) which is a sum-score of the following items: P1 delusions, P2 conceptual disorganization, P3 hallucinations, N1 blunted affect, N4 social withdrawal, N6 lack of spontaneity and flow of conversation. Each item ranged from 1 to 7 points, with a total score ranging from 6 to 42 points.

#### 2.1.4 Statistics

All statistics were performed using SPSS (version 28.0). Demographic and clinical data are described using means and standard deviations (SD) or absolute and relative frequencies depending on the type of variable.

For further analyses of the 31 patients a multi-step analytical approach was applied: Since the CSF/serum albumin ratio is a key parameter describing inflammatory influences in psychotic disorders at the border (BCSFB) of the periphery and the CNS, this parameter was used as the main CSF measure (Campana et al., 2024). Sex was coded as a binary variable, with men coded as 1 and women coded as 0.

In a first exploratory step, Spearman's rank correlations were calculated between (1) the CSF/serum albumin ratio (2) the pLGI score, (3) age (4) PANSS6 sum score, (5) GAF score and (6) social isolation sum score to estimate the bivariate associations. Additionally, Mann- Whitney U tests were conducted to assess sex differences in the above-mentioned variables.

Furthermore, a correlation matrix including the raw values of the four parameters contributing to the pLGI score, as well as the CSF/serum albumin ratio was calculated to provide further insight into the interrelationships among the components of the score.

In a second step, a multiple linear regression analysis was conducted with the CSF/serum albumin ratio as dependent variable and the remaining six variables from step one as predictors in order to assess the unique contribution of each predictor while controlling for the others.

A sensitivity analysis was performed by re-running the regression after excluding influential observations with a Cook's distance greater than 4/N (=0.129).

Based on the set of six potential predictors and the complete sample (N = 31), a forward stepwise regression procedure was applied to identify the most relevant predictors (inclusion criterion, p ≤ 0.05; exclusion criterion p ≥ 0.10).

In addition, two extended regression models were estimated: one including all two-way interactions among the three predictors, and a full model additionally including the three-way interaction. Continuous predictors were mean-centered prior to computing interaction terms, whereas dichotomous variables were not centered. Given the limited sample size, models including interaction terms were interpreted with caution.

All relevant assumptions for multiple linear regression (linearity, independence of errors, homoscedasticity, normality of residuals, and absence of multicollinearity) were tested and found to be met.

Differences were considered statistically significant at p < 0.05. Due to the exploratory nature of the study, no adjustments for multiple comparisons were applied.

## 3 Results

### 3.1 Demographic and clinical findings

In total 53 cases with lumbar puncture diagnostic were screened. 31 of these patients had either a SSD or affective psychosis and a CRP score <10 mg/l (to rule out an acute inflammatory state) upon admission and were therefore included in the analysis (main demographic, diagnostic, serum and CSF findings are presented in Table 1A).

**Table 1:** **A)** Presentation of main demographic and clinical findings. **B)** Correlation analysis: Spearman's Correlation of different parameters. pLGI score. PANSS6. GAF. CSF/serum albumin ratio. Age in years. Social isolation score. pLGI score: (r = 0.418, p = 0.019), Age in years (r = 0.415, p = 0.020). **C)** Correlation analysis: Spearman's Correlation of different parameters of the pLGI. Platelets (r = 0.490, p = 0.005). **D)** Regression analysis: Summary of multiple linear regression analyses with the CSF/serum albumin ratio as the dependent variable. Model 1 comprises the full model with all predefined predictors. Models 2–4 reflect successive steps of a forward stepwise regression approach, resulting in a final model including pLGI score, age, and sex. Note. Sex was coded as 0 = female, 1 = male. p values < 0.05 were considered statistically significant. ∗: p < 0.05. ∗∗: p < 0.01. CRP: C-reactive Protein. CSF: cerebrospinal fluid. EEG: Electroencephalography. FLAIR: fluid-attenuated inversion recovery. GAF: Global Assessment of Functioning. G/L ratio: granulocyte-to-lymphocyte ratio. MRI: cranial magnetic resonance imaging. N: number. PANSS6: short form of the Positive and Negative Syndrome Scale. pLGI: peripheral low-grade inflammation. SD: Standard Deviation. WBC: white blood cell count.

Table 1A – Clinical and social characteristics		
	N	%
**Clinical/Social Background**		
Male	15/31	≈48.0
Female	16/31	≈51.0
F20 (Schizophrenia)	16/31	≈51.0
F21 (Schizotypal disorder)	1/31	≈3.2
F23 (Acute polymorphic psychotic disorder)	5/31	≈16.0
F25 (Schizoaffective disorder)	5/31	≈16.0
F32.3 (Severe depressive episode with psychotic symptoms)	3/31	≈9.6
F33.3 (Recurrent depressive disorder, current episode severe with psychotic symptoms)	1/31	≈3.2
Previous antipsychotic medication	13/31	≈42.0 out of this ≈85.0% atypical ≈15.0% typical
Smoker	15/31	≈48.0
Legal care	3/31	≈9.6
Assisted living	3/31	≈9.6
Background of migration	14/31	≈44.8
Somatic diseases (single or multiple)	7/31	≈22.5
- Circulatory	2/31	≈6.4
- Diabetes	2/31	≈6.4
- Thyroid gland	2/31	≈6.4
- Neurological	1/31	≈3.2
- Respiratory	2/31	≈6.4
- HIV	1/31	≈3.2
- Musculoskeletal	0/31	≈0.0
- Cancer	0/31	≈0.0
Urine toxicology pathologies/substance use (4 with cannabis, 1 with alcohol - no signs of substance-induced psychosis)	5/31	≈16.0
EEG pathologies (1 with intermitted slowing, 1 with sharp waves, 1 with signs of higher excitability)	3/31	≈9.6
Cranial MRI pathologies (2 unspecific FLAIR lesions, 1 with unspecific T2 lesions)	3/31	≈9.6

**Time Duration**	**mean**	**SD**
Age in years (range 18 to 66, N = 31)	38.7	±13.3
Duration of disease in years (N = 31)	3.7	±4.9

**Diagnostic**		
GAF (range 0-100%, N = 31)	25.3	±7.6
PANSS6 (range 6-42, N = 31)	21.6	±6.4
- item delusions	3.5	±2.0
- item conceptual disorganization	4.0	±1.4
- item hallucinations	2.3	±1.8
- item blunted affect	4.0	±1.7
- item social withdrawal	4.5	±1.9
- item lack of spontaneity and flow of conversation	3.4	±1.6
pLGI score (range −16 to +16, N = 31)	−0.5	±7.1
- WBC	−0.1	±2.5
- CRP	−0.1	±2.5
- Platelet count	−0.2	±2.5
- G/L ratio	−0.1	±2.5
Social Isolation score (range 0-4, N = 31)	2.0	±1.2

**Blood (N = 31)**		
CRP upon admission	2.2 mg/l	±2.3
WBC count upon admission	6.8/μl	±1.9
Platelet count upon admission	250/nl	±60.1
Neutrophil count upon admission	4.1/nl	±1.6
Basophil count upon admission	0.01/nl	±0.0
Eosinophil count upon admission	0.2/nl	±0.1
Lymphocyte count upon admission	1.9/nl	±0.6
Granulocyte count upon admission	4.3/nl	±1.7
G/L ratio upon admission	2.5	±1.6
Albumin serum (35-53 g/l)	43.8	±3.9

**CSF (N = 31)**		
Leucocyte count (range 0-4/μl)	2.8 cells	±2.1
- Pleocytosis ≈22.5% (7/31)		
Protein (range 150-450 mg/dl)	429 mg/dl	±249
Albumin (<340 mg/dl)	270 mg/dl	±171
CSF/serum albumin ratio (<6.7)	6.0	±3.7
- CSF/serum albumin ratio out of range ≈ 22.5% (7/31)		

Table 1B: Correlation analysis							
*Correlation analysis*		CSF/serum albumin ratio	pLGI score	Age in years	PANSS6	GAF	Social isolation score
**CSF/serum albumin ratio**	Correlation coefficient	1.000					
	P two sided						
	N	31					
**pLGI score**	Correlation coefficient	**0.418∗**	1.000				
	P two sided	**0.019**					
	N	**31**	31				
**Age in years**	Correlation coefficient	**0.415∗**	0.131	1.000			
	P two sided	**0.020**	0.484				
	N	**31**	31	31			
**PANSS6**	Correlation coefficient	0.130	−0.123	0.160	1.000		
	P two sided	0.487	0.510	0.390			
	N	31	31	31	31		
**GAF**	Correlation coefficient	−0.349	−0.326	−0.301	−0.267	1.000	
	P two sided	0.054	0.073	0.100	0.146		
	N	31	31	31	31	31	
**Social isolation score**	Correlation coefficient	−0.134	−0.334	0.001	0.215	0.063	1.000
	P two sided	0.472	0.066	0.997	0.245	0.738	
	N	31	31	31	31	31	31

Table 1C: Correlation analysis of pLGI single items						
*Correlation analysis*		CSF/serum albumin ratio	WBC	CRP	Platelet count	G/L ratio
**CSF/serum albumin ratio**	Correlation coefficient	1.000				
	P two sided					
	N	31				
WBC	Correlation coefficient	0.275	1.000			
	P two sided	0.134				
	N	31	31			
**CRP**	Correlation coefficient	0.058	0.322	1.000		
	P two sided	0.757	0.077			
	N	31	31	31		
**Platelet count**	Correlation coefficient	**0.490∗∗**	**0.479∗∗**	0.069	1.000	
	P two sided	**0.005**	**0.006**	0.713		
	N	**31**	**31**	31	31	
G/L **ratio**	Correlation coefficient	0.305	**0.592∗∗**	−0.025	0.261	1.000
	P two sided	0.096	**0.000**	0.892	0.156	
	N	31	**31**	31	31	31

Table 1D: Regression analysis						
*Model*	*Predictor*	Regression Coefficient	Standard error	Beta Coefficient	T-values	P two sided
**1**	**pLGI score**	0.173	0.091	0.335	1.903	0.069
	**Sex**	2.293	1.140	0.316	2.012	0.056
	**Age in years**	0.096	0.045	0.347	2.124	**0.044**
	**PANSS6**	0.075	0.097	0.131	0.776	0.446
	**GAF**	−0.031	0.087	−0.064	−0.355	0.726
	**Social isolation score**	−0.232	0.529	−0.074	−0.438	0.665
**2**	**pLGI score**	0.217	0.087	0.418	2.481	**0.019**
**3**	**pLGI score**	0.192	0.082	0.371	2.336	**0.027**
	**Age in years**	0.101	0.044	0.367	2.313	**0.028**
**4**	**pLGI score**	0.186	0.078	0.359	2.394	**0.024**
	**Age in years**	0.106	0.041	0.383	2.552	**0.017**
	**Sex**	2.251	1.080	0.311	2.085	**0.047**

CSF/serum albumin ratio as dependent variable.

According to ICD-10 (ICD-10-GM, 2020) we excluded non-psychotic patients: 4 with F33.2 (Recurrent depressive disorder, current episode severe without psychotic symptoms), 5 with F32.2 (Severe depressive episode without psychotic symptoms), 1 with F31.6 (Bipolar affective disorder, current episode mixed), 1 with F42.2 (Mixed obsessional thoughts and acts), 1 with F45.0 (Somatization disorder), 1 with F60.3 (Emotionally unstable personality disorder) and 1 with F03 (Unspecified dementia). Further, we excluded 2 patients with F20.0 (Schizophrenia), 2 F25.0 (Schizoaffective disorder) and 1 F23.1 (Acute polymorphic psychotic disorder with symptoms of schizophrenia) due to CRP >10 mg/l upon admission and 1 patient with F20 because of missing further diagnostic data. 1 case of neurosyphilis and 1 case of probable autoimmune psychosis with mGluR1 antibodies in serum was excluded (Graus et al., 2016; Pollak et al., 2020; Herken et al., 2017). The 5 patients to be excluded due to CRP values above 10 mg/l presented with CRP values between 20.0 and 62.5 mg/l upon admission and most had competing reasons to low-grade inflammatory findings like among others cold symptoms, hypothermia, condition following smoke inhalation and bruise due to car accident.

For the included 31 patients the mean age was 38.7 years (±13.3 SD) and mean duration of disease was 3.7 years (±4.9 SD). Mean pLGI score (range −16 to +16) was −0.5 (±7.1 SD). Mean PANSS6 sum score (range 6 to 42) was 21.6 (±6.4 SD). The social isolation score (range minimum 0 points (indicating low social isolation) to maximum 4 points (indicating high social isolation)) revealed a mean of 2.0 (±1.1 SD). Mean GAF was 25.3 (±7.6 SD). Mean CSF/serum albumin ratio was 6.0 (±3.7 SD). Positive anti-neuronal antibodies in serum were found in ≈12.8% (4/31; Recoverin antibodies and Purkinje cell binding, Neuropil antibodies, GFAP antibodies with fluorescence of subpial astrocytes and CASPR2 antibodies) and 0.0% (0/31) in CSF. Positive AI were found in ≈12.8% (4/31; but negative PCR in serum and CSF). Positive OCBs were found in ≈12.8% (4/31; 2 weak positive in CSF and serum; 2 positive in CSF).

### 3.2 Correlations

In our analysis, we observed significant correlations between the CSF/serum albumin ratio and the pLGI score (r = 0.418, p = 0.019), as well as with age (r = 0.415, p = 0.020). There also was a negative correlation showing a trend toward significance for the GAF score (r = −0.349, p = 0.054). No other correlation reached a level of significance (Table 1 B). Fig. 1B illustrates the correlation between the pLGI score and the CSF/serum albumin ratio. Mann-Whitney-U-test did not indicate significant sex differences for the above-mentioned variables (p > 0.1).

The correlation between the four parameters contributing to the pLGI score, and the CSF/serum albumin ratio shows a significant correlation between platelets and the CSF/serum albumin ratio (r = 0.490, p = 0.005) but no significant correlation between G/L ratio and CSF/serum albumin ratio (r = 0.305, p = 0.096), whereas CRP and WBC did not show a significant correlation with CSF/serum albumin ratio (p > 0.1). Table 1C, correlation analysis of pLGI single items sums up the respective correlation matrix to additionally illustrate, how individual parameters are correlated with each other.

### 3.3 Multiple linear regression

A multiple linear regression analysis was conducted to examine the association between six predictors (age, sex, pLGI score, PANSS6 sum score, GAF score and social isolation sum score) and CSF/serum albumin ratio as outcome variable. The overall model was statistically significant (F(6,24) = 3.002, p = 0.025) explaining a moderate proportion of variance (adjusted R^2^ = 0.286). Among the predictors, age shows a significant association with the outcome (p = 0.044) whereas sex (p = 0.056) and pLGI score (p = 0.069) showed trend-level association.

Sensitivity analysis excluding four influential cases (Cook's distance >0.129) confirmed model significance and slightly improved variance explanation (F(6,20) = 3.308, p = 0.020; adjusted R^2^ = 0.348) along with marginally improved regression coefficients for the respective predictors (data not shown).

Forward stepwise regression identified pLGI score as the first significant predictor (F(1,29) = 6.154, p = 0.019; adjusted R^2^ = 0.147). Adding age improved model fit (F(2,28) = 6.213, p = 0.006; adjusted R^2^ = 0.258), and inclusion of sex in the final model explained 33.7% of the variance (F(3,27) = 6.087, p = 0.003; adjusted R^2^ = 0.337).

Table 1D presents the regression coefficients, standard errors, standardized beta coefficients, t-values and significance levels for each predictor across the four regression models.

Inclusion of two-way and three-way interaction terms did not reveal significant interactions (p > 0.2) and reduced explained variance (data not shown).

## 4 Discussion

As the main finding, our study demonstrates a significant positive correlation between a measure of BCSFB permeability, i.e. the CSF/serum albumin ratio, and the pLGI score. Interestingly, the main parameter driving the pLGI score were platelets. Further, the BCSFB permeability positively correlated with patients’ age. Additionally, the regression model supports the unique impact of age, sex and pLGI score on the CSF/serum albumin ratio. Hereby, male sex predicts higher values for the CSF/serum albumin ratio.

Sensitivity analyses excluding influential cases confirmed the stability of the regression model, as exclusion did not reduce model fit and slightly increased the explained variance and regression coefficients. Moreover, inclusion of two- and three-way interaction terms did not improve model fit, indicating that age, sex, and pLGI score primarily exert independent effects on CSF/serum albumin ratio.

Albumin is the main part of the total protein in the CSF and the CSF/serum albumin ratio is commonly used as an indicator of BCSFB permeability (Reiber, 1980). Additionally, BCSFB permeability, as described by our results, has been found to increase with age (Tumani et al., 2017) and has been described in neurological diseases (Parrado-Fernandez et al., 2018), dementia (Musaeus et al., 2020), bipolar disorder (Zetterberg et al., 2014) and depression (Sorensen et al., 2022). As described previously the evidence for a higher CSF/serum albumin ratio in psychotic disordered is a robust finding compared to controls and even individually matched healthy controls age 18-50 years (Orlovska-Waast et al., 2019) (Jeppesen et al., 2022). 22,5% of our patients had CSF/serum albumin ratio above reference level. Other studies found 29,4% abnormal rates among 331 patients (Oviedo-Salcedo et al., 2021 and 24,4% among 531 patients (Campana et al., 2024) and are in line with our findings.

Increased CSF/serum albumin ratios have been reported for male psychotic patients and higher symptoms scores (Campana et al., 2024) (Meixensberger et al., 2020). A reason in male patients could be related to comorbidities (Meixensberger et al., 2020) (Pollak et al., 2018) and longer spine in men (Reiber, 1994), which could affect outflow of CSF out of the spinal nerve (Bechter et al., 2014) (Benveniste et al., 2015). Further, protective hormonal factors (Maggioli et al., 2016) and a response to antipsychotic treatment were discussed in females (Rabinowitz et al., 2014). In our study we could not find correlations with the PANSS6 score which could be due to the retrospective design but a trend for the GAF score. Nevertheless, our analysis goes one step further by demonstrating that the CSF/serum albumin ratio in our cohort is related to the pLGI score.

We excluded patients with CRP above 10 mg/l upon admission to rule out an acute inflammatory state or influence of infectious comorbidities according to the pLGI score but the cut-off of 10 mg/l for CRP values previously has been discussed critically to differentiate between acute and chronic inflammatory influences (Mac Giollabhui et al., 2020). Other studies used different CRP levels e.g. for patients with depression to focus on functional connectivity alterations due to inflammation (CRP < 2 mg/l vs CRP > 2 mg/l) (Bekhbat et al., 2022). Consistent with data on somatic diseases, our cohort was moderately ill (22.4%) (Campana et al., 2022). The exact value of peripheral inflammatory findings in psychotic disorders and the mechanism of influence on central nervous structures remains unclear. Interestingly, peripheral inflammatory markers seemed not to correlate with CSF inflammatory markers in paired blood-CSF samples (Gigase et al., 2023).

CRP has been discussed to be a state and trait marker in schizophrenia (Lestra et al., 2022), and experimental data point towards a possible BBB disruption (Kuhlmann et al., 2009). However, peripheral CRP levels were not related to CSF alterations, raising questions about whether peripheral inflammation, like an increase in CRP, is leading to BCSFB alteration and inflammation in the CSF in schizophrenia (Campana et al., 2022). Interestingly, WBC, neutrophil, basophil, eosinophil and monocyte count were reported to be increased in patients with psychotic disorders (Jeppesen et al., 2022) (Nunez et al., 2019) (Steiner et al., 2020) (Llorca-Bofi et al., 2024) (Obeagu et al., 2024) and this may be influenced by antipsychotic treatment (Stefanovic et al., 2015).

BBB damages by peripheral inflammation can be divided into disruptive and non-disruptive mechanisms. Disruptive mechanisms include among others modification of tight junctions via matrix metalloproteinases (Qin et al., 2015), endothelial damage and apoptosis via mitogen-activated protein kinase (Karahashi et al., 2009) and astrocyte damage (Asgari et al., 2015) as well as non-disruptive mechanisms including modifications of transporters for amino acids (Wittmann et al., 2015) and beta amyloid (Jaeger et al., 2009), cerebral endothelial activation by IL-1β and TNF-α (Skelly et al., 2013) and cellular transmigration (Bohatschek et al., 2001) (Wang et al., 2008). A BBB leakage was found in SSD using MRI techniques (Moussiopoulou et al., 2025). Most important, the other way around it was discussed that in diseases of the brain the BBB may be more vulnerable to systemic inflammation (Varatharaj et al., 2017). Compared to the BBB, the BCSFB permeability was also studied before but only few literature is discussing effects of peripheral inflammation on the morphology of the BCSFB in psychosis including relations of the choroid plexus volume to monocyte counts and high-sensitive CRP and IL-6 (Lizano et al., 2019) (Majerova et al., 2025) (Yakimov et al., 2024b). In other diseases such as multiple sclerosis tight-junctions pathologies including loss of claudin-3 in the choroid plexus have been reported post-mortem (Kooij et al., 2014) and BCSFB disruption due to matrix metalloproteinase 3 in Alzheimer's disease model (Brkic et al., 2015). More general the choroid plexus is discussed to be a hub of immune activity following acute brain inflammation and peripheral inflammatory stimulus recruiting immune cells to the choroid plexus from the brain and the periphery and inducing cytokines like pro-inflammatory TNF-α and IL-1β mRNA (Marques et al., 2007) (Xu et al., 2024)**.**

It needs to be mentioned that among all parameters of the pLGI score, higher platelet counts correlated with higher BCSFB permeability in our study. Mean platelet volume was discussed to be increased in schizophrenia (Cabello-Rangel et al., 2023) and an elevation of platelets in antipsychotic-treated patients correlated significantly with aripiprazole, ziprasidone and haloperidol. Higher platelet counts correlate with WBC and are associated with non-responders and the PANSS-negative subscale (Zhang et al., 2024). Platelets can express dopamine receptors and take up dopamine, which makes them interesting in cases of psychotic disorders (Ehrlich et al., 2012). Moreover, platelet aggregation in patients with schizophrenia differs from that in healthy controls (Dietrich-Muszalska et al., 2009).

Platelets are thought to play a dual role in the BBB protection via clot formation but also a disruptive role via inflammatory mechanisms. Platelets can release factors like P-selectin, platelet-activation factors, platelet-derived growth factors and Amyloid-β (Aβ) (Lv et al., 2023) (Carrano et al., 2011) (Wolska et al., 2023). In Alzheimer's disease Aβ was discussed to wrap vessels and breakdown BBB (Wisniewski et al., 1997). The mechanism is discussed to be neuroinflammatory mediated since Aβ is a toxic protein leading to downregulation of tight junctions between endothelia cells via claudin-5 and involves NOX-2-positive activated microglia (Carrano et al., 2011). Further, it was discussed that Aβ can bind to NMDA receptors of endothelial cells leading to activation of protein kinase C, induction of Ca2+ influx and then influencing BBB integrity (Shi et al., 2010). Further, platelets can release TNF-α, IL-10 and interleukin 1β mediating neuroinflammation related to the BBB (Theofilis et al., 2021). Interestingly, platelet-derived growth factor beta and its receptor loss in knockout mice are discussed to be essential for a loss of pericytes and BBB breakdown which could be a further mechanism (Bell et al., 2023). Today, compared to the BBB mentioned above, effects of platelets on the BCSFB are rare. A role of platelet activation markers and CRP aggregates were discussed to be related to the choroid plexus in post-mortem samples of patients with amyotrophic lateral sclerosis (Saul, 2020). It can be discussed that similar effects of platelets on the BCSFB might be possible as mentioned above for the BBB since BCSFB also includes tight junctions between epithelial cells of the choroid plexus and platelet-derived growth factor which can be secreted by endothelial cells, epithelial cells and platelets as well. Taken together, platelets have a multifaceted role beyond their role in hemostasis (Burnouf and Walker, 2022).

Using a more comprehensive approach, the pLGI score in our study correlates with the CSF/serum albumin ratio and is a useful tool integrating different peripheral inflammatory findings (CRP, WBC, platelet count, G/L ratio) of the body in one score. We cannot conclude that the proposed model is leading to inflammation of the CNS. In schizophrenia scores like neutrophil to lymphocyte ratio (NLR), monocyte to lymphocyte ratio (MLR), platelet to lymphocyte ratio (PLR), and the systemic inflammation index (SII) were discussed to be cost effective, reproducible and less affected by other factors compared to absolute cell counts (Mojadadi et al., 2024) (Sandberg et al., 2021) (Brinn et al., 2020) (Bhikram et al., 2022) (Bioque et al., 2022).

The social isolation score failed to moderate our proposed model of peripheral inflammation related to BCSFB permeability which could be reflected by the retrospective design. Further, it is possible that social isolation is an objective measure, but that the subjective experience of loneliness could be stronger to affect mental health (Wang et al., 2017). Evidence suggests an interrelation between social interaction and inflammation in psychosis (Faustmann et al., 2023a), but the feeling of loneliness seems to be more relevant than social isolation, as seen in depression (Gao et al., 2024). Loneliness significantly correlates with psychosis (Michalska da Rocha et al., 2018) but patients do not always feel alone when social isolated or even feel alone when surrounded by others underlining that the self-related feelings are more important in this context and should be addressed (Campagne, 2019; Cacioppo et al., 2016). Recently, data in chronically socially stressed rodents and humans point towards a stress-induced myeloid cell activation influencing the BBB and further central nervous structures, such as the nucleus accumbens, via matrix metalloproteinase 8 in depression (Cathomas et al., 2024).

## 5 Limitations

Clearly, our analysis has limitations: Social interaction difficulties should be explored more comprehensively both in terms of subjective and objective measures in future studies. This is a small, retrospective, monocentric pilot study, and the integration of inflammation, social interaction and psychotic disorders is complex (Faustmann et al., 2023a). Especially, the results of the multiple linear regression should be interpreted with caution due to the small sample size. Due to exploratory nature of the study, no alpha level adjustment was used. Somatic disorders and medication should be considered when interpreting the results, as they may have influenced the inflammatory results, but findings are presented by a natural cohort from the western part of Germany. However, we focused on low CRP values to exclude acute and severe inflammatory conditions. To the best of our knowledge, our study is the first to integrate blood, CSF and social parameters of psychotic disorders in a retrospective exploratory approach to shed new light on these interactions. Future studies should apply further hypothetically driven approaches to e.g. reduce problems of multiple comparison and to confirm these findings and should increase sample size to enhance power for analyses.

## 6 Conclusion

The pLGI score appears to be useful for the evaluation of inflammation in psychotic disorders and appears to be linked to measures of BCSFB permeability. Platelets are interesting parameters related to BCSFB permeability in SSD in this study. Future studies are needed to confirm this and to investigate links with other CSF parameters. If confirmed, the pLGI score could be used to recommend lumbar puncture in absence of signs for autoimmune psychosis since it is easy to assess and linked to BCSFB disruption. Further, social isolation is highly objective. Future studies should involve a differentiate measure of social behavior using the evaluation of subjective loneliness e.g. the UCLA-Loneliness questionnaire and further digital techniques like e.g. camera- and smartphone-based motion tracking techniques (Russell et al., 1980) (Lahnakoski et al., 2020) (Sahandi Far et al., 2021). Using a prospective design, future studies should investigate differentiate analysis of immunological parameters of blood and CSF together with measurements of social behavior (Faustmann et al., 2023a). What exact mechanism platelets have on the BCSFB/BBB in schizophrenia needs to be further determined.

## CRediT authorship contribution statement

**Timo Jendrik Faustmann:** Writing – review & editing, Writing – original draft, Validation, Project administration, Methodology, Formal analysis, Data curation, Conceptualization. **Aykut Aytulun:** Writing – review & editing, Validation, Methodology, Data curation. **Armin Bahic:** Writing – review & editing. **Michaela Jänner:** Writing – review & editing, Software. **Leonhard Schilbach:** Writing – review & editing, Supervision. **Daniel Kamp:** Writing – review & editing, Visualization, Validation, Software, Methodology, Formal analysis.

## Ethics approval

The study was approved by the ethics committee of the Medical Faculty of Heinrich-Heine-University (study number 2023-2513).

## Availability of data and materials

All relevant data are included in the manuscript.

## Funding

Does not apply.

## Declaration of competing interest

The authors declare that they have no known competing financial interests or personal relationships that could have appeared to influence the work reported in this paper.

## Data Availability

Data will be made available on request.
